# Detection of *Rickettsia raoultii* in *Vermipsylla alakurt*-Like Fleas of Sheep in Northwestern China

**DOI:** 10.1007/s11686-024-00809-y

**Published:** 2024-02-28

**Authors:** Fengshi Li, Shanshan Zhao, Ente Li, Songsong Xie, Nan Wang, Wenbo Tan, Yuanzhi Wang

**Affiliations:** 1 Key Laboratory for Prevention and Control of Emerging Infectious Diseases and Public Health Security, the XPCC, School of Medicine, Shihezi University, Shihezi City, 832002 Xinjiang Uygur Autonomous Region People’s Republic of China; 2 The First Affiliated Hospital of Shihezi University Medical School, Shihezi City, 832002 Xinjiang Uygur Autonomous Region People’s Republic of China

**Keywords:** *Rickettsia raoutii*, PCR, Vermipsyllidae, Fleas, Sheep, Northwestern China

## Abstract

**Introduction:**

To date, a total of 2574 validated flea species have been discovered. Vermipsyllidae is a family of fleas that comprises at least eight species. *Vermipsylla* is a genus of the family Vermipsyllidae within the order Siphonaptera of fleas. Here a novel *Vermipsylla* species was described, and rickettsial agent was also detected in it.

**Methods:**

A total of 128 fleas were collected directly from 260 pastured sheep in China. Of these, eight representative fleas (four males and four females) were identified by key morphological features. Meanwhile, 120 flea DNAs, including six flea samples for molecular taxonomy, were subjected to *Rickettsia* spp. DNA detection. The molecular identity of fleas was determined by amplification and sequenmce analysis of four genetic markers (the *28S* rDNA genes, the *18S* rDNA genes, the mitochondrial cytochrome c oxidase subunit I and subunit II). In addition, five *Rickettsia*-specific gene fragments were used to identify the species of the rickettsial agents. The amplified products were sequenced and phylogenetically analyzed.

**Results:**

The morphological characteristics of the flea species identified in this study were similar to *Vermipsylla alakurt*, but presented difference in hair number of the metepimeron, the third tergum, the genitals and the tibiae of hind leg. The *18S* rDNA, *28S* rDNA and CO*II* genetic markers from fleas showed the highest identity to those of *V*. *alakurt*, shared 98.45% (954/969), 95.81% (892/931) and 85.86% (571/665) similarities, respectively. However, the *COI* sequence showed the highest identity to that of *Dorcadia ioffi* with 88.48% (576/651) similarity*. Rickettsia raoutii* tested positive in 14.17% (17/120) flea DNA samples.

**Conclusion:**

Our study reports the detection of *R. raoultii* in *V. alakurt*-like fleas infesting sheep in China.

## Introduction

Fleas (Insecta: Siphonaptera) are small, laterally flattened, wingless, and highly specialised insects [1], which belong to arthropod phylum, insecta, Siphonaptera. Currently, at least 2575 validated flea species belonging to 16 families and 246 genera have been described [2]. *Vermipsylla*, a genus of the family Vermipsyllidae, includes eight validated species, i.e. *Vermipsylla alakurt* (Kazakhstan, Mongolia, China), *V*. *asymmetrica* (China), *V*. *ibexa* (China), *V*. *minuta* (China), *V*. *parallela* (China), *V*. *perplexa* (China, Nepal), *V*. *quilianensis* (China) and *V*. *yeae* (China) [3, 4]. *V*. *alakurt* was firstly identified in China in 1965, in the southern region of Xinjiang Uygur Autonomous Region (XUAR), northwestern China [5]. During December to March, the adult fleas are mainly endemic in alpine pastoral areas, and prevailingly infest sheep, yaks, horses, yellow cattle and some wildlife species, causing irritation, poor condition, anaemia, abortion and even death [6–8].

Fleas are of tremendous medical and economic importance as vectors of several diseases important to human health including bubonic plague, murine typhus, and epidemic typhus [9, 10]. *Rickettsia typhi* in *Ctenocephalides felis*, *Rickettsia* *felis* in *Liposcelis bostrychophila* and Candidatus* Rickettsia barbariae* in *V*. *alakurt*, were previously reported [11–13].

## Materials and Methods

### Sample Collection and Identification of Fleas

In January 2018, fleas (128 in total) were collected directly from the entire body of 260 pastured sheep from two sheep flocks in Altaw Mountain, Wenquan County (the north region of XUAR, 2200 m a.s.l; 44°470ʹ30 N, 80°53ʹ30 E), which was adjacent to Kazakhstan [14]. The collected fleas were divided into two parts. Eight representative fleas (four males and four females) were for morphological identification by Stereomicroscope according to key features [13, 15] (eg. body length, labial palpus and notch of the tibiae of hind leg). In addition, DNAs of the individual fleas were extracted using the TIANamp Genomic DNA Kit (TIANGEN, Beijing, China) according to the manufacturer’s instructions. Six DNA from six representative flea samples were subjected to PCR amplification of four genetic markers [the *28S* rDNA gene, the *18S* rDNA gene and the mitochondrial cytochrome c oxidase subunit I (*COI*) and subunit II (CO*II*)] for molecular identification. The nucleotide sequence were manually edited and compared to GenBank reference sequences (http://www.ncbi.nlm.nih.gov/BLAST/). Phylogenetic trees were constructed by the MEGA 7.0 software with the Maximum Likelihood (ML) method [16].

### Detection of Rickettsial Agents and Sequence Analysis

A total of 120 flea DNAs, including six flea samples for molecular taxonomy, were subjected to PCR amplification for the detection of *Rickettsia* spp. DNA. Five rickettsial genetic markers, 17-kilodalton antigen 17-kilodalton antigen (*17-kDa*), surface cell antigen 4 (*sca4*), citrate synthetase (*gltA*), surface cell antigen 1 (*sca1*), and outer membrane proteins A (*ompA*) were used according to published protocols [17, 18]. Each PCR assay included a negative control (distilled water instead of flea DNA template) and a positive control (DNA from Candidatus* R*. *barbariae* obtained from *V*. *alakurt*). The above procedures were applied to treat the PCR products and their corresponding sequences. A phylogenetic tree was constructed by the MEGA 7.0 software with the ML method [13].

## Results

**Fig. 1:**
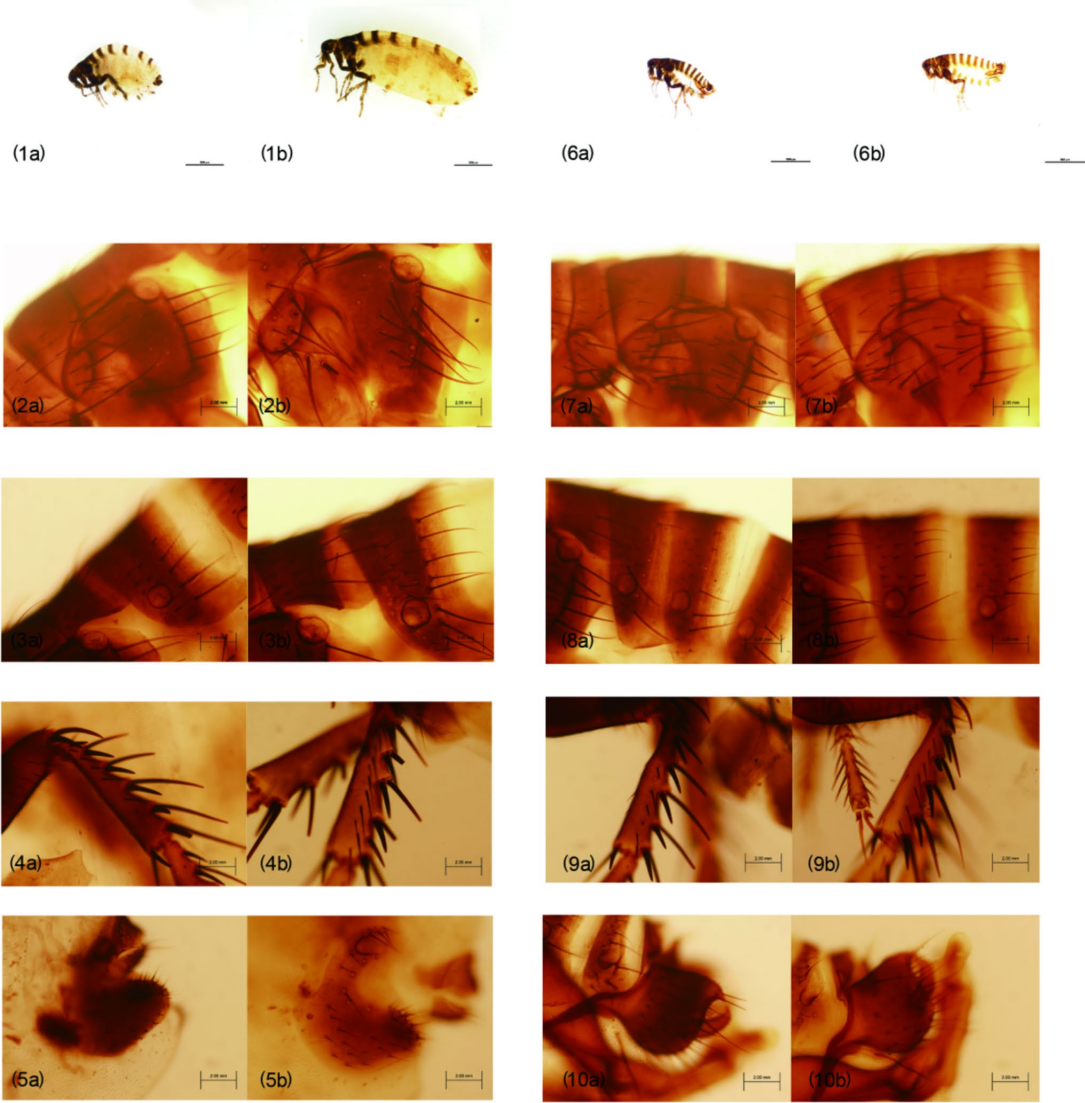
Photomicrographs of *V. alakurt*-like and *V. alakurt.* 1a: the whole body of *V. alakurt*-like, female*.* 1b: the whole body of *V. alakurt*, female*.* 2a: the metepimeron of *V. alakurt*-like, female*.* 2b: the metepimeron of *V. alakurt*, female*.* 3a: the third tergum of *V. alakurt*-like, female*.* 3b: the third tergum of *V. alakurt*, female*.* 4a: the tibiae leg of *V. alakurt*-like, female*.* 4b: the tibiae leg of *V. alakurt*, female*.* 5a: the spermathecae of *V. alakurt*-like, female*.* 5b: the spermathecae of *V. alakurt*, female*.* 6a: the whole body of *V. alakurt*-like, male*.* 6b: the whole body of *V. alakurt*, male*.* 7a: the metepimeron of *V. alakurt*-like, male*.*7b: the metepimeron of *V. alakurt*, male*.* 8a: the third and fourth tergum of *V. alakurt*-like, male*.* 8b: the third and fourth tergum of *V. alakurt*, male*.* 9a: the tibiae leg of *V. alakurt*-like, male*.* 9b: the tibiae leg of *V. alakurt*, male*.* 10a: the aedoeagus of *V. alakurt*-like, male*.* 10b: the aedoeagus of *V. alakurt*, male

A total of 128 fleas were collected from the entire body of 260 sheep (10 males, 250 females) in two flocks in Altaw Mountain, Wenquan County, which is adjacent to Kazakhstan [14]. The fleas were divided into two parts: eight fleas (four males and four females) were preserved for morphological identification, and the remains were used for other purposes. The collected fleas had the following morphological characteristics similar to *V*. *alakurt*, which can be clearly distinguished from the other seven *Vermipsylla* species (shown in Table 1). Its size was the largest in the members of *Vermipsylla* genus, with males measuring 3.7–4.9 mm and females measuring 5.6–7.5 mm or longer. Its labial palpus was no longer than 17 segments, with 12–15 segments in females and 10–14 segments in males. The rear part of the female flea was full of fat, making up about three-quarters of its body length. The head of the intromittent organ of the males looked like a winter glove (with the back four fingers held together). The head of the spermathecae is ellipsoid, and the tail part was thin and long, with a sausage-like shape. The above morphological characteristics was similar to *V*. *alakurt*, interestingly, the females had less hairs (*n* = 5) next to the notch of the tibiae of hind leg, which obviously distinguishes from *V*. *alakurt* (*n* = 13). The differences from other key morphological features, eg. the hair of the metepimeron, the hair of the third tergum, the hair of the tibiae of hind leg and the hair of the genitals, were shown in Fig. 1 and Table 2. Due to the limited data available in GenBank, only two Vermipsyllidae species, *V*. *alakurt* and *Dorcadia ioffi*, were compared. The sequenced PCR product of *18S* rDNA, *28S* rDNA and *COII* obtained from fleas in this study were 98.45% (954/969), 95.81% (892/931) and 85.86% (571/665) similarities to *V*. *alakurt* (GenBank accession number KR297206, KR297207, KT193612), respectively. However, the *COI* nucleotide sequence of fleas in this study were 91.16% identical to *D. ioffi* in GenBank (accession number MF124314). Based on morphological and molecular evidence, all the fleas were identified as a novel *Vermipsylla* species, here named as *Vermipsylla alakurt*-like.

**Table 1:** The difference in the host, geographical distribution, clasper, aedoeagus, spermathecae, mesopodium,metapodium,the seventh sternum, the eighth sternum, the number of labial palp and the body length among *V*. *asymmetrica, V*. *minuta, V*. *parallela, V*. *ibexa, V*. *yeae, V*. *quilianensis, V*. *perplexa* and *V*. *alakurt*

	Host	Geographical distribution	Clasper	Aedoeagus	Spermathecae	Mesopodium	Metapodium	The Seventh sternum	The Eighth sternum	Labial palp	Body length
*V. asymmetrica*	Musk deer	Tibet, Qinghai (China)	Length than width, with a longitudinal ossification rod	Basically divided into two lobes	The head is rounder and slightly larger than the eyes	Asymmetry of the position of the second contralateral metatarsal mane of the fifth toe segment	The third and fourth tarsus are particularly flattened, with dense bristle clusters on both sides, and the fifth toe is long	The ventral margin has a reflexed ossified sheet	/	11–12	♂: 2.2–3.2 mm ♀: 3.1–10.7 mm
*V*. *minuta*	Wild ass	Sichuan (China)	Dorsal leaf is large, terminal is apiculate, lateral leaf is posterior, with beak shaped ossifying hook process	/	/	The third and fourth toe segments are triangular	There is a tuft of bristle before cutting the posterior margin of the first phalangeal node	Only a list of bristle	End projecting posteriorly, triangular or conical	♂: 9–12 ♀: 11–15	♂: 1.8–2.1 mm ♀: 2.2–3.2 mm
*V*. *parallela*	Cattle, yak, Yunnan snub-nosed monkey	Tibet, Yunnan (China)	/	The posterior furrow has a sparse mane, and the length of the bristle is made up near the end of the immobile process, whose end is far below the movable process	The tail has a mastoid process	The first toe segment has a bunch of bristles	/	Usually with 2 list of bristles	The end is round and convex	10–12	♂: 3.0–3.3 mm ♀: 3.5–5.5 mm
*V*. *ibexa*	Ibex	Xinjiang (China)	Be similar to oval in shape	Lower half significantly wider than upper half, immobile protruding and movable protruding from the middle of posterior margin. Stalk process club-shaped, mostly parallel on both sides	Head round and short tail	The bristle of the fifth toe is symmetrical	/	/	The number of bristles under the eighth midplane valve is more in females, about 20	10–11:	♂: 2.0–4.2 mm ♀: 2.0–2.3 mm
*V*. *yeae*	Sheep	Xinjiang (China)	/	Dorsal margin not rounded and convex	/	Oval in shape, tail grows longer than head, caudal end has ossified mastoid process	/	/	/	10–12	♂: 4 mm ♀: 6 mm
*V*. *quilianensis*	Red deer, deer	Gansu, Qinghai (China)	Anterior margin proximal to slightly concave The outer bristle of the body is less (10–15)	Dorsal leaves slender, lateral leaves peachy, heavily ossified oblique ridges protruding from end margin	/	The third and fourth toe segment of the middle hind foot is triangular	/	/	/	9–12	♂: 3.0–3.9 mm ♀: 5.0–7.0
*V*. *perplexa*	Goral, goat, sheep, roe deer, musk deer	Tibet, Qinghai (China)	The number of lateral bristles and lower lip whisker segments is significantly less than the named subspecies	The immobile process is beak shaped	The head is oval and less than the width of the eyes	The third and fourth toed segment is triangular. No mane cluster	/	A steamed bread shaped	/	/	♂: 2.6–3.9 mm ♀: 2.3–4.9 mm
*V*. *alakurt*	Sheep, yak, horse, goat, cow, red deer	Gansu, Qinghai, Xinjiang, (China), Kazakhstan, Kyrgyzstan, Mongolia	/	Like as winter gloves	Head rounded or nearly rounded	/	/	/	/	♂: 10–14 ♀: 12–15	♂: 3.7–4.9 mm ♀: 5.6–7.5 mm

**Table 2:** The difference in the amount of hair and bristle among *V. alakurt* (male), *V. alakurt*-like (male), *V. alakurt* (female) and *V. alakurt*-like (female) in metepimeron, the third tergum, the fourth tergum, tibiae leg, aedoeagus and spermathecae

	Metepimeron		The third tergum		Tibiae leg	Aedoeagus		Spermathecae	
	Bristle	Hair	Bristle	Hair	Hair	Long hair	Short hair	Long hair	Short hair
*V. alakurt,* male	*n* = 10	*n* = 11	*n* = 7	*n* = 14	*n* = 9	*n* = 17	*n* = 18	/	/
*V.alakurt*-like, male	*n* = 10	*n* = 15	*n* = 6	*n* = 17	*n* = 6	*n* = 18	*n* = 20	/	/
*V. alakurt*, female	*n* = 10	*n* = 10	*n* = 9	*n* = 21	*n* = 11	/	/	*n* = 13	*n* = 30
*V. alakurt*-like, female	*n* = 10	*n* = 13	*n* = 6	*n* = 13	*n* = 7	/	/	*n* = 10	*n* = 16

**Fig. 2:**
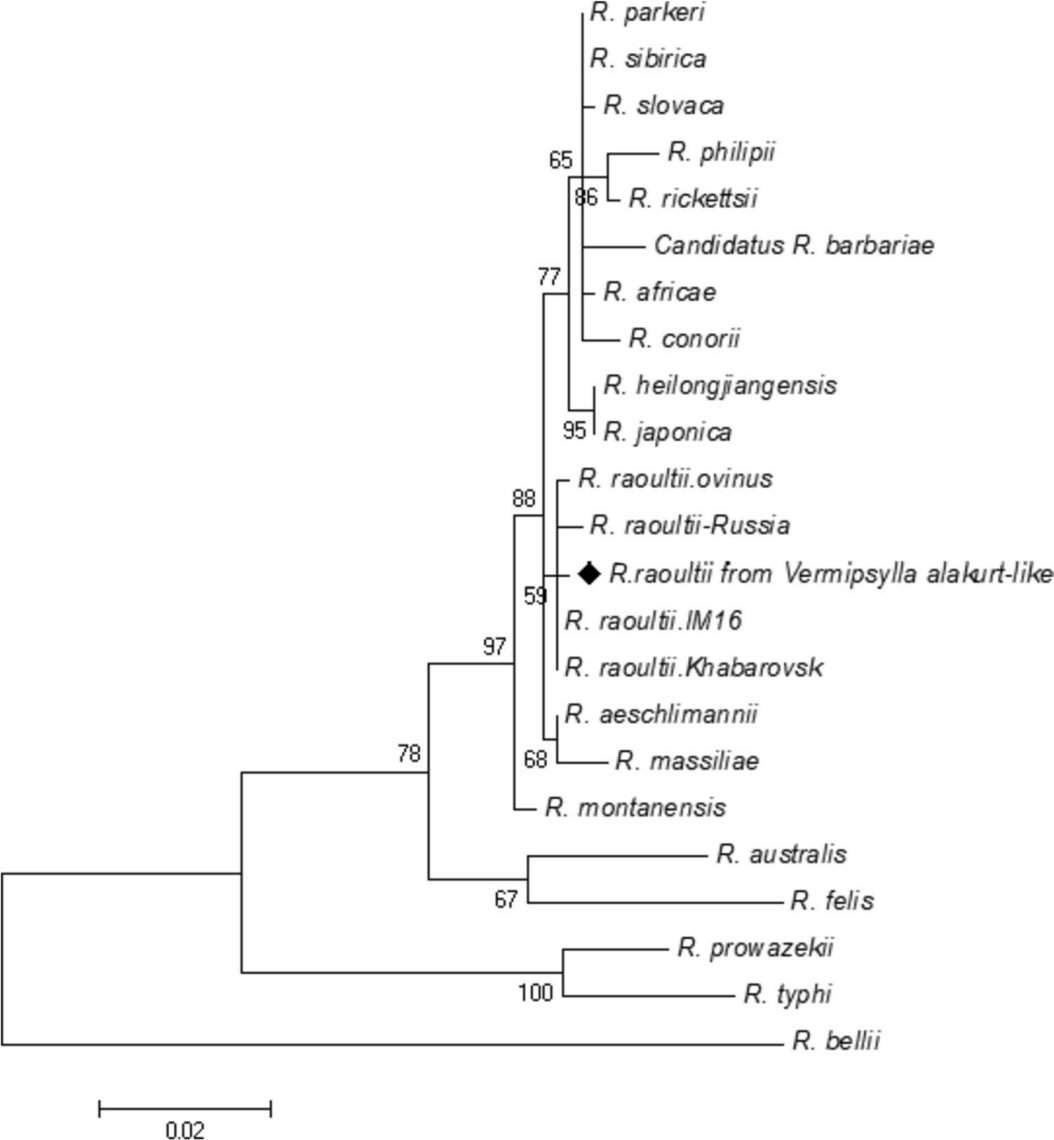
Maximum-likelihood (ML; 500 bootstrap replicates) phylogenetic tree of the *17*-*kDa*-*ompA*-*sca4*-*gltA-sca1* constructed with MEGA7, using the sequences of *R. raoultii* from *V. alakurt* -like (◆) in this study and sequences from *Rickettsia* species retrieved from the GenBank database. The sequences for *R. bellii* were used as an outgroup. The scale bar represents the inferred substitutions per nucleotide site. The relative support for clades in the tree produced from the ML and NJ analyses are indicated above and below the branches, respectively

Seventeen out of 120 flea DNA samples screened for *Rickettsia* spp. DNA were positive for the five genetic markers (*17-kDa*, *ompA*, *sca4*, *gltA* and *sca1*). No nucleic acids were amplified from the negative controls. The six responsive genetic markers, namely *17-kDa*, *gltA*, *ompA*, *sca4*, and *sca1* exhibited high sequence similarities with the genome of *Rickettsia raoultii* strain Khabarovsk (CP010969): 99.44% (360/362), 99.89% (896/897), 99.32% (441/444), 100% (762/762), and 99.64% (549/551), respectively. The phylogenetic tree revealed that *R*. *raoultii* was confirmed in this study (shown in Fig. 2).

All of the obtained sequences were deposited in GenBank [*17-kDa*: MZ449221, *gltA*: OP376870, *ompA*: OP376871, *sca4*: OP376872, *sca1*: OP376873, *18S* rDNA: OP339795, *COI*: OP324573, *28S* rDNA: OP339754, *COII*: OP433454].

## Discussion

Combined with molecular identity, systematic morphological identification in arthropods is important for the identification of a new species or subspecies [19, 20]. To date, morphology can also be used as a helpful tool to distinguish flea species or subspecies, such as *C*. *felis* and *C*. *canis* [21]. The flea species identified in this study can be classified into the *Vermipsylla* genus according to some morphological key features, such as no combs, its labial palpus less than 17 segments, the tibiae of hind leg having 6 notches, full of fat at the rear 3/4 part of the female body, only one seminal vesicle and its head being ellipsoid [22]. Although CO*I* of *Vermipsylla alakurt*-like shared higher similarity (88.48%) with *D. ioffi* than datum between *Vermipsylla alakurt*-like and *V*. *alakurt*, we still believed *Vermipsylla alakurt*-like presents its own characteristics. Interestingly, *Vermipsylla alakurt*-like and *V*. *alakurt* have some similarities, such as segment number (*n* = 10–15) of labial palpus, the aedoeagus (in the shape of a winter glove) and the spermathecae (the head is ellipsoidal, and the tail is slender and sausage-shaped). Meanwhile, there are also some differences, especially in the number of hair and bristle of metepimeron, the third tergum, the tibiae of hind leg, aedoeagus and spermathecae (shown in Table 2), which makes it distinct from the other eight validated species of *Vermipsylla* genus, including *V*. *alakurt*. This finding indicates that the morphological characteristics (eg. the hair number of the metepimeron, the third tergum, the genitals and the tibiae of hind leg) could be helpful in identification and taxonomy especially for *Vermipsylla* species.

In this study, we firstly confirmed *R*. *raoultii* was detected in a novel *Vermipsylla* species. To date, *R*. *raoultii* have been detected in a sheep ked and 20 tick species, namely, *Melophagus ovinus*, *Dermacentor nuttallii*, *De*. *marginatus*, *De*. *reticulatus*, *De*. *silvarum*, *Rhipicephalus pumilio*, *Rh*. *sanguineus*, *Rh*. *annulatus*, *Ixodes persulcatus*, *I*. *ricinus*, *I*. *canisuga*, *I*. *kaiseri*, *Haemaphysalis longicornis*, *Ha*. *erinacei*, *Ha*. *Punctata*, *Ha*. *concinna*, *Ha*. *japonica*, *Amblyomma helvolum*, *Hyalomma asiaticum*, *Hy*. *anatolicum* and *Hy*. *marginatum* [23–37]. Previously, *R*. *raoultii* was rarely reported in fleas exception of *C*. *felis* [38]. This is the first time that *R*. *raoultii* has been found in Vermipsyllidae.

Although the vast of the spotted fever group rickettsiae (SFGR) are transmitted by ticks or sheep keds [23, 39], there are exceptions. *R*. *africae*, *R*. *felis*, Candidatus *R*. *barbariae*, belonging to the members of SFGR, were also detected in *C*. *garei* fleas (from passerine birds that had migrated from Africa) [40], *C*. *felis* fleas (collected from sheep, cats and dogs) [41] and *V*. *alakurt* (from sheep), respectively [13]. Herein, we reported the presence of *R*. *raoultii* in *Vermipsylla alakurt*-like fleas from sheep in an alpine pastoral area in the north-western of China. There is not enough evidence to confirm that *Vermipsylla alakurt*-like can transmit rickettsiosis to sheep. In future, detecting *R. raoultii* in sheep organs would demonstrate its potential to cause the disease.

Previously, the special geographical environment and current research confirms that *R*. *raoultii* is highly prevalent in the northwest of China and its neighboring countries, such as Mongolia, Russia and Kazakhstan [22, 42, 43]. We should pay attention to the transmission of SFGR in a wider arthropod species especially in the blood-suckers.

## Conclusion

A novel *Vermipsylla* species from alpine pastoral region was described. This is the first report of the presence of *R*. *raoultii* in Vermipsyllidae. These findings extend our knowledge of *Vermipsylla* species, and the geographical distribution and reservoir hosts for *R*. *raoultii*. Future research should assess the vector competence and transmission dynamics of *Vermipsylla alakurt*-like for *R. raoultii* and other rickettsial pathogens.

## Data Availability

The datasets used and/or analyzed during the present study are available from the corresponding author on reasonable request.
